# Lemmel’s Syndrome: A Report of Two Cases at the Hospital General Dr. Manuel Gea Gonzalez in Mexico City

**DOI:** 10.7759/cureus.38378

**Published:** 2023-05-01

**Authors:** Ana Priscila Campollo Lopez, Luis Eduardo Cárdenas Lailson, Fernando Barbosa Villarreal, Mauricio Gutierrez-Alvarez, Adolfo Cuendis Velázquez, Alejandro Cruz Zarate

**Affiliations:** 1 General Surgery, Hospital General Dr. Manuel Gea González, Mexico, MEX

**Keywords:** duodenal diverticula, periampullary duodenal diverticula, endoscopic retrograde cholangiopancreatography (ercp), jaundice cholestatic, surgical case reports, lemmel’s syndrome

## Abstract

The term ¨Lemmel Syndrome¨ is used to describe obstructive jaundice that is secondary to periampullary duodenal diverticula (PDD) in the absence of choledocholithiasis or neoplasia. PDD is found in 22% of the population. According to our knowledge, only two cases of Lemmel syndrome have been reported in Mexico. We report two cases of Lemmel syndrome in a 94-year-old and a 71-year-old woman who presented with clinical jaundice. One of the cases was treated with endoscopic retrograde cholangiopancreatography (ERCP) sphincterotomy, balloon sweep, and the placement of a plastic biliary prosthesis, and the other with laparoscopic biliodigestive bypass and a manual lateral end choledocho-duodenal anastomosis. Our objective is to expand the information on this rare pathology to take it into account as a diagnostic possibility of jaundice and to define appropriate management, which can be endoscopic or surgical.

## Introduction

Lemmel syndrome (LS) was first described by Dr. Gerhard Lemmel in 1934 [1,2] and is defined as obstructive jaundice secondary to periampullary duodenal diverticula (PDD) in the absence of choledocholithiasis or neoplasia [3]. The PDDs are typically located at 2 or 3 cm from the ampulla of Vater [2], they are found in 22% of the population, and less than 10% are symptomatic [1,2,4], causing pancreaticobiliary complications (gallbladder and bile duct gallstones, cholangitis, acute or chronic pancreatitis) and non-pancreaticobiliary complications (hemorrhage, duodenocolic fistula, perforation, enterolithiasis) [4].

## Case presentation

Clinical case 1

A 94-year-old woman has undergone an open cholecystectomy and arterial hypertension treated with captopril as part of her important medical history. She presented to the emergency department for medical evaluation, complaining of localized pain in the epigastrium after four days of evolution, associated with nausea, vomiting of gastro-alimentary content on multiple occasions, fever, jaundice, and choluria.

Upon physical examination, there was mucocutaneous jaundice. Abdominal tenderness in the mesogastrium, without visceral or palpable masses. Vital signs were a blood pressure of 128/85 mmHg, a heart rate of 74 bpm, a respiratory rate of 19 bpm, SaO2 of 98%, and a temperature of 37.6ºC. Her blood workup results are shown in Table 1.

**Table 1:** Summary of important findings on complete blood count, liver function tests and pancreatic enzyme tests. * alanine transaminase **aspartate aminotransferase

	Value	Reference values	Measurement unit
Leucocytes	16,3	4,5 to 10,0	μl
Neutrophils	12	1,53 to 7,4	μl
Platelets	56	150 to 400	μl
Total bilirubin	7.68	0.3 to 1	mg/dL
Direct bilirubin	5.12	0.1 to 0.3	mg/dL
ALT*	272	7 to 56	U/L
AST**	195	5 to 40	U/L
Amylase	1,444	60 to 180	U/L
Lipase	168	10 to 140	U/L

An abdominal ultrasound was requested as part of the diagnostic work-up, and the findings were: the common bile duct was 1.1 cm, the pancreas had inflammatory data, the gallbladder was surgically absent, and the portal vein was 1.6 cm.

**Figure 1:**
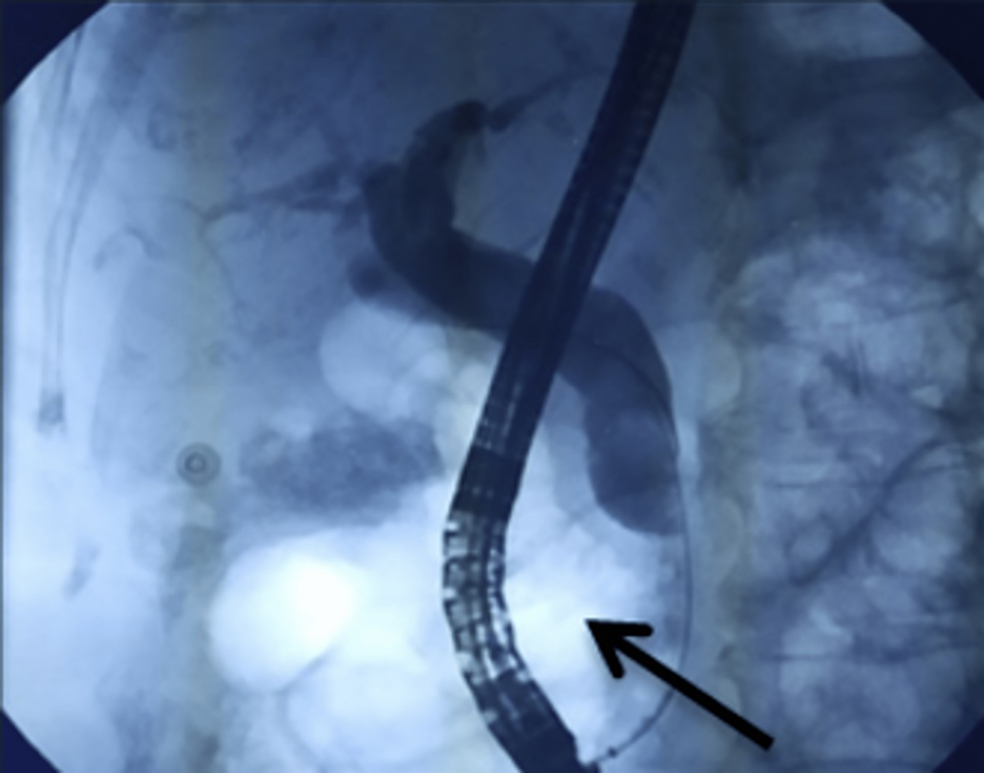
Cholangiography shows a dilated bile duct associated with the presence of a diverticulum, indicated with a black arrow.

**Figure 2:**
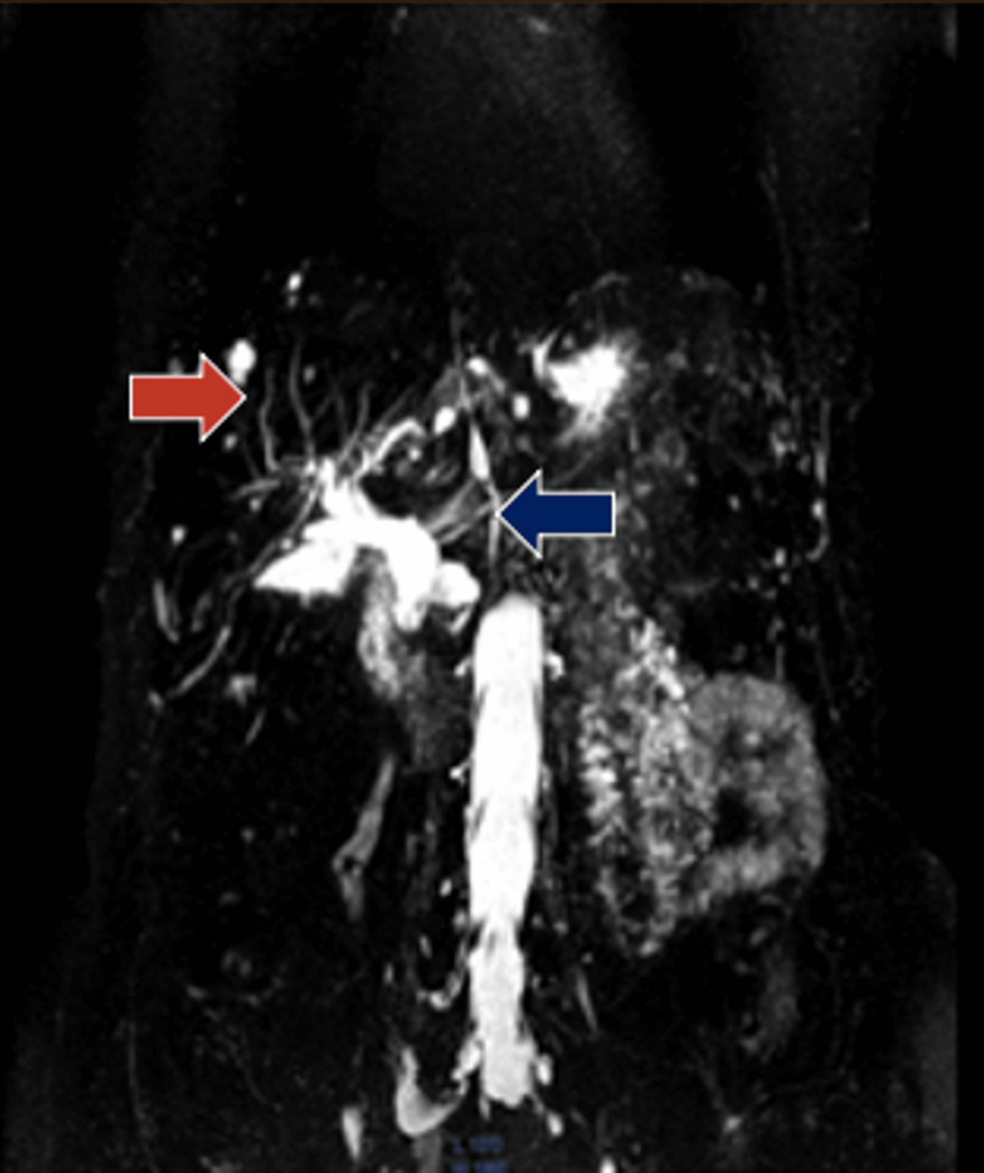
Magnetic resonance cholangiopancreatography shows a dilated extrahepatic bile duct without choledocholithiasis (blue arrow) and the absence of a gallbladder (red arrow).

**Figure 3:**
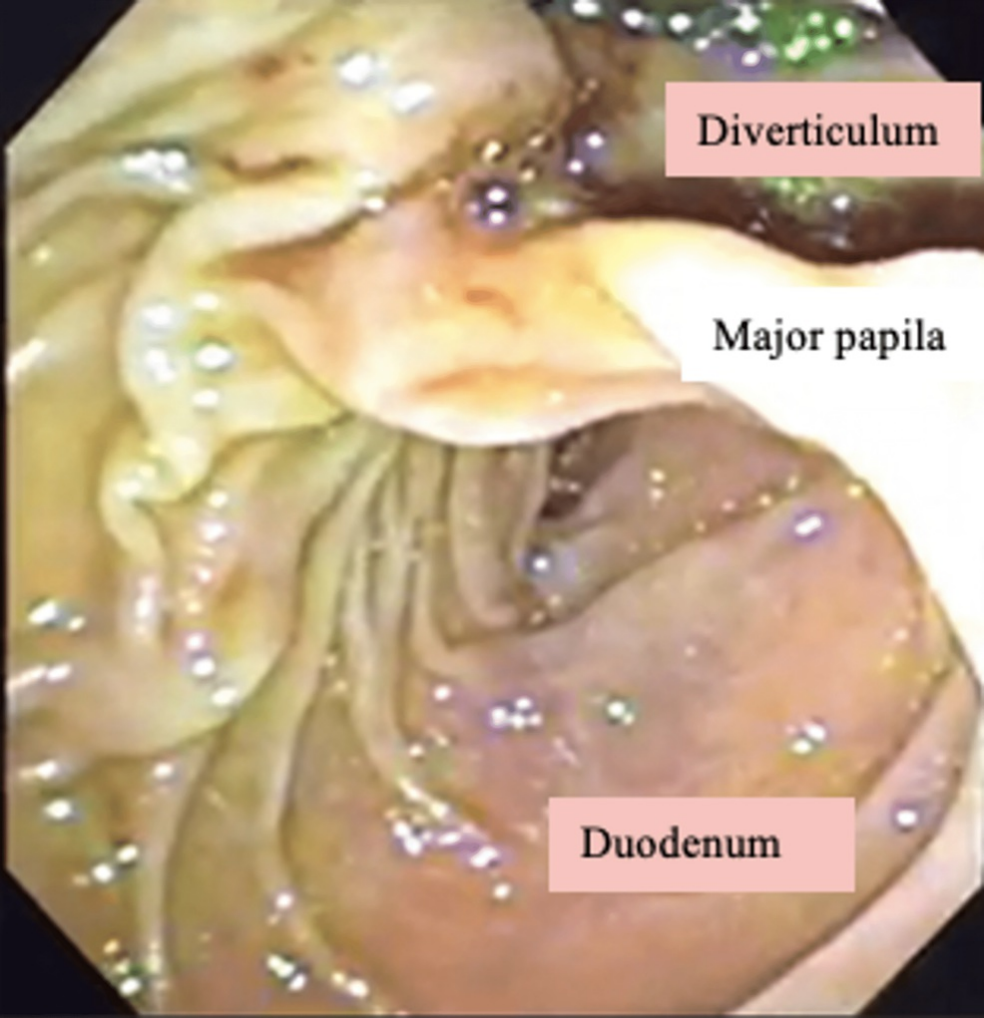
The duodenum and a giant juxtapapillary duodenal diverticulum are shown in the ERCP.

The patient was admitted to the general surgery department with a diagnosis of moderate-severe acute cholangitis and acute pancreatitis. Cholangiography and magnetic resonance cholangiopancreatography (MRCP) was requested as part of the diagnostic approach (Figures 1, 2), showing a dilated bile duct associated with the presence of a diverticulum. An emergency endoscopic retrograde cholangiopancreatography (ERCP) was performed, which revealed purulent cholangitis, a giant juxtapapillary duodenal diverticulum, a probable peri-diverticular fistulous orifice, and a dilated extrahepatic biliary tract (Figure 3). It was performed with a sphincterotomy, balloon sweep, and placement of a 10 Fr x 10 cm plastic biliary prosthesis without any complications. The patient improved clinically and was discharged eight days after ERCP.

Clinical case 2

A 71-year-old woman, whom, as part of her important medical history, she refers to as having been diabetic and having hypothyroidism. She was hospitalized due to cholangitis and choledocholithiasis, and an ERCP was performed, reporting unresolved choledocholithiasis, so stent placement was performed. One month later, the patient was readmitted with moderate acute cholangitis, for which a new ERCP was performed. During the new ERCP, an Amsterdam biliary prosthesis of 7 Fr x 10 cm was placed due to a previously migrated biliary prosthesis. Following this, balloon sweeping with gallstone extraction and laparoscopic cholecystectomy was performed.

She returned to the emergency room of our hospital seven months later, reporting abdominal pain localized in the epigastrium, abdominal distension, nausea, and subjective fever, predominantly in the evening, for four days. Vital signs were blood pressure at 126/82 mmHg, heart rate at 72 bpm, respiratory rate at 17 bpm, SaO2 at 96%, and temperature at 36.9ºC. Physical examination revealed scleral icterus staining, a painful abdomen on palpation in the epigastrium and right hypochondrium, without visceromegaly or palpable masses, and no evidence of peritoneal irritation.

Her blood workup results were as shown in Table 2. An abdominal CT scan was performed, revealing a selective right-sided stent with a distal end before the intrapancreatic segment, dilatation of the left intrahepatic bile duct, pneumobilia, surgical absence of the gallbladder, dilatation of the pancreatic duct, and uncomplicated diverticular disease. Due to this reason, she was admitted to the general and endoscopic surgery departments with a diagnosis of moderate acute cholangitis.

**Table 2:** Summary of important findings on complete blood count and liver function tests. * alanine transaminase **aspartate aminotransferase ***international normalized ratio

	Value	Reference values	Measurement units
Leucocytes	14,9	4,5 - 10,0	μl
Neutrophils	13,6	1,53 - 7,4	μl
Platelets	128	150 - 400	μl
Total bilirubin	1.2	0.3 - 1	mg/dL
Direct bilirubin	0.9	0.1 - 0.3	mg/dL
ALT*	54	7 - 56	U/L
AST**	38	5 - 40	U/L
Alkaline phosphatase	130	44 – 147	U/L
Gamma glutamyl transpeptidase	159	5 – 40	U/L
Albumin	3.2	3.4 - 5.4	g/dL
INR***	1.27	0.50 – 1.90	

A new ERCP was performed where Vater's ampulla was identified with data from previous sphincterotomies, as well as juxtapapillary diverticulum, biliary prosthesis migrated to the right hepatic duct, dilated intrahepatic biliary tract, common hepatic duct of 16.3 mm, and permeable cystic duct, so a biliary endoprosthesis was placed and a balloon sweep was performed. A laparoscopic biliodigestive bypass and a manual lateral end choledocho-duodenal anastomosis were scheduled for her (video 1). After a favorable development, the patient was discharged and continued as an outpatient for follow-up.

**Video 1:** Laparoscopic lateral end choledocho-duodenal. Laparoscopic repair is observed, starting with the posterior wall of the choledochus and intestine.

## Discussion

The prevalence of periampullary duodenal diverticula (PDD) is very low in the general population. In Mexico, only two cases of Lemmel syndrome (LS) have been reported [5,6]. PDDs are usually asymptomatic, but they may present as right upper quadrant pain with elevated bilirubin, transaminases, and pancreatic enzymes, simulating choledocholithiasis or pancreatitis [1], as in the case of our two patients who were misdiagnosed at their first approach. According to Love JS et al., in a review of 16 case reports of LS, the most common symptom was pain (64.7%), followed by fever (52.9%). The diagnosis is made by exclusion or an incidental finding. As part of the diagnostic approach, blood tests can be requested to reveal elevations in serum total bilirubin in 80% of the cases and elevations in liver enzymes in 70% of the cases [2]. Studies such as CT or magnetic resonance cholangiopancreatography (MRCP) have shown the presence of periampullary diverticula, but endoscopic retrograde cholangiopancreatography (ERCP) has diagnostic and therapeutic value [1].

The objectives of the treatment of LS are to decrease pancreato-biliary complications secondary to bile duct obstruction [5]. Conservative or surgical treatment is only needed for PDD that is symptomatic because its complications, although rare, are very serious. For biliopancreatic and hemorrhagic complications, endoscopic treatment is the first-line treatment. Indirect surgical treatment involves bilioenteric bypass or duodenal exclusion, while direct surgical treatment involves diverticulectomy [7].

When endoscopic or conservative treatment fails, surgical treatment is usually reserved [8]. Endoscopic sphincterotomy is the initial treatment for biliopancreatic complications of PDDs and is successful in 95% of cases. The presence of PDDs makes it difficult to cannulate, but the range of failed cannulations is low, between 2% and 4% [5]. If an endoscopic sphincterotomy is not feasible, bilioenteric bypass is preferred over diverticulectomy, as this procedure has a high risk of morbidity and mortality [7,9]. Diverticulectomy is associated with a high risk of injury to the bile and pancreatic ducts. The retroperitoneal location of this second duodenal part makes surgical procedures in this part complex [8]. The risk of duodenal fistula is high and is associated with a mortality rate of 20% to 30% [7].

The biliodigestive bypass procedure is easy to perform and is associated with morbidity of 1% to 8% and mortality of less than 6%, which are lower than diverticulectomy. The biliodigestive bypass is an effective treatment for additional symptoms in most cases, but the patient remains at risk for recurrent pancreatitis as the bypass does not treat the sphincter dysfunction caused by the impingement of the diverticulum on the pancreatic duct. The laterolateral choledochoduodenostomy is the simplest and quickest procedure, especially in adults with multiple comorbidities [7].

## Conclusions

As a cause of biliary obstruction, Lemmel syndrome is a very rare clinical entity. Therefore, it is important to consider it as a differential diagnosis for proper management. The treatment of a patient with symptomatic Lemmel syndrome can be either endoscopic or surgical and is based on the clinical characteristics of the patient and the outcome of in-hospital management.
